# Associations of *SLC6A20* genetic polymorphisms with Hirschsprung’s disease in a Southern Chinese population

**DOI:** 10.1042/BSR20182290

**Published:** 2019-08-13

**Authors:** Xiaoli Xie, Qiuming He, Lihua Huang, Le Li, Yuxiao Yao, Huimin Xia, Jinglu Zhao, Wei Zhong, Yan Zhang

**Affiliations:** Department of Pediatric Surgery, Guangzhou Institute of Pediatrics, Guangzhou Women and Children’s Medical Center, Guangzhou Medical University, Guangzhou 510623, Guangdong, China

**Keywords:** Hirschsprung’s disease, SLC6A20, Southern Chinese

## Abstract

Hirschsprung’s disease (HSCR) is a neurodevelopmental disorder characterized by the absence of nerves in intestine with strong genetic components. *SLC6A20* was found to be associated with HSCR in Korean population waiting for replication in an independent cohort. In the present study, ten single nucleotide polymorphisms (SNPs) in the *SLC6A20* were selected from Southern Chinese with 1470 HSCR cases and 1473 ethnically matched healthy controls. Our results indicated that SNP rs7640009 was associated with HSCR and *SLC6A20* has a gene–dose effect in the extent of the aganglionic segment during enteric nervous system (ENS) development. It is the first time to reveal the relationship between SNP rs2191026 and HSCR-associated enterocolitis (HAEC) susceptibility.

## Introduction

Hirschsprung’s disease (HSCR) is a congenital disease characterized by the absence of intrinsic ganglion cells in the submucosal and myenteric plexuses of the intestinal tract involving a variable portion. It is common that HSCR leads to the mechanical intestinal obstruction in infants and children. At present, surgery is a unique way to cure HSCR in spite of its fraught complications such as constipation, diarrhea, incontinence and so on. If left untreated, HSCR can lead to death. Similar to Western Europe [1] and North America [2], the incidence in Asians is approximately 1 in 5000 neonates [3,4]. HSCR was classified by the length of intestine lacking nerve cells, known as short-segment HSCR (SHSCR) when the aganglionic segment does not extend beyond the upper sigmoid, long-segment HSCR (LHSCR) when the aganglionosis extends proximally to the sigmoid, or total colonic aganglionosis (TCA) when the aganglionosis extends throughout the entire colon [5]. To the most extreme, total intestinal aganglionosis (TIA), whose aganglionosis extends to almost total intestines in sporadic rare cases, can be deadly [6]. While all cases present a functional intestinal obstruction, but the mode of presentations often differs. The diagnosis of HSCR is mostly made in the newborn period, only a few patients’ diagnoses are delayed into infancy stage or adulthood. The typical clinical features are as follows: delayed passage of meconium (24 h after birth), constipation in the neonatal period, abdominal distension and vomiting during the first few days of life and neonatal enterocolitis. Persistent constipation, chronic abdominal distension, loss of appetite and failure to thrive are common symptoms in older children [5]. Timely surgical treatment to remove the affected bowel segment can save most patients’ lives, but obvious postoperative complications, such as fecal incontinence, constipation, repeated enterocolitis, nutrition disorder and so on [7], could bring the patients and their families severe economic burden and great psychological pressure [8], which might affect their quality of life in adulthood [9,10]. Even more, severe complications like HSCR-related short bowel syndrome could also kill the patients after surgery [11].

It is widely believed that genetic factors playing an important role though the etiology of HSCR is still unknown. In the past decades, several genes have been identified to be associated with HSCR, including *RET* [12], *EDNRB* [13], *GDNF* [14] and *SOX10* [15], which however failed to explain the disease cause of large sporadic cases. In 2014, *SLC6A20* on chromosome 3p21.3 was suggested as associated with HSCR using 124 patients and 450 controls [16]. These single nucleotide polymorphisms (SNPs) were subsequently replicated, and 13 SNPs were significantly associated with HSCR using 187 patients and 283 unaffected controls [17]. The haplotype analysis also indicated their significant associations with HSCR susceptibility. Limited by the sample size and incomplete clinical records in previous studies, the functional mechanisms of the variants related to the identified HSCR were not clarified. In addition, it is still necessary to further validate the associations between these SNPs derived from one single causal variant or the congregation of multiple variants that is still awaited for further validation in independent cohorts.

In order to further investigate the association between *SLC6A20* and HSCR susceptibility, a case–control study was conducted to verify the effects of selected SNPs from an independent South Chinese population with 1470 cases and 1473 controls. We failed to further verify the association between nine previously confirmed SNPs and HSCR except SNP rs7640009. However, six of the other SNPs were found to contribute to the risk of severe subtypes of HSCR in the following detailed analysis stratified by clinical subphenotypes. Furthermore, the relationships between selected SNPs and HSCR-associated enterocolitis (HAEC) susceptibility were also examined.

## Materials and methods

### Study population

From 2005 to 2015, 1470 HSCR patients from Guangzhou Women and Children’ Medical Center were selected in our study, including 1034 SHSCR, 295 LHSCR, 82 TCA and 3 TIA patients. Inclusion: (1.) sporadic HSCR with histological examination of biopsy specimens with the absence of the enteric ganglia; (2.) age <18 years; (3.) claimed as south Chinese. Exclusion: diagnosis of familial HSCR or syndromic HSCR. The blood samples of 1473 individuals matched geographically and ethnically with no history of HSCR or neurologically associated disorders were collected as controls. Written informed consent was obtained from all the subjects and the legal guardians of every child. The study protocol conformed to the ethical guidelines of the 1975 Declaration of Helsinki and was approved by the Ethics Committee on Human Research of the Faculty of Guangzhou Women and Children’ Medical Center.

### SNP genotyping and quality control

Nine SNPs (rs2191027, rs6770261, rs2191026, rs10461016, rs4299518, rs764177, rs2108917, rs17279465 and rs78962450) of *SLC6A20* involved in the present study were selected from to the replication study with 187 Korean HSCR cases [17]. Rs4299518 in *SLC6A20* was chosen from the GWAS of HSCR by Kim et al. [16]. Ten SNPs in *SLC6A20* were genotyped by MassARRAY iPLEX Gold system (Sequenom) on all the samples. The associated studies searched by NCBI based on the criteria as follows: (1.) SNP with high probability to be regulatory variants were kept for further pursuing (Regulome DB score higher than 2. http://regulomedb.org/). (2.) Removed one of the two SNPs with Linkage disequilibrium (LD) (*r^2^*) larger than 0.8 were kept one. (3.) SNP with minor allele frequency larger than 5% in the Chinese population (Han Chinese in Beijing (CHB)) was kept (https://www.ncbi.nlm.nih.gov/snp/?term=). We carried out quality control of the ten SNPs using the following two criteria: (1.) SNPs with >10% missing data were removed (one SNP). (2.)Three SNPs were removed according to the genotyping allele intensity plots for clustering quality and violation of Hardy–Weinberg equilibrium (HWE). SNPs were removed if HWE, *P*<1.0E-04, was calculated by control subjects. After quality control, all ten SNPs were kept for further analysis consisted of 1470 cases and 1473 controls).

### Association analysis and subphenotype analysis

The SNPs were analyzed for associations with the disease using a comparison of the minor allele frequency in patients and controls (basic allelic test) as well as other tests using PLINK1.9 (genotype test of 3 × 2 contingency tables, Cochran–Armitage trend test, a test of dominant and recessive models). Association of the SNPs with disease risk was also corrected by logistic regression using age and sex as covariates and the associations found in the present study remain significant. Association with subphenotype was analyzed by comparing cases with a certain subphenotype with controls, cases without the subphenotype with controls.

### Independence testing

LD patterns and values were obtained using HaploView. SNPTEST v2.5b was used to perform the logistic regression tests in the present study. Tests of independent contributions toward disease associations for SNPs in a single locus were done using logistic regression, adjusting for the effect of a specific SNP (COVsnp) in the same locus. Stepwise logistic regression was performed by SPSS 16.0. Briefly, variables were added to the logistic regression equation one at a time, using the statistical criterion of reducing the 2Log10 Likelihood error for the included variables. After each variable was entered, each of the included variables was tested to see whether the model would be better off if the variable were excluded.

### Haplotype analysis

First, all founders are phased using the E-M algorithm implanted in PLINK, which will generate haplotype-specific tests (1df) for both disease and quantitative traits; an omnibus association statistic will also be computed (P_omnibus). In all cases, the tests were based on the expected number of haplotypes each had. Then the association was performed on all the most likely haplotype assignments as SNPs and used all the standard analytic options (P). If there are H haplotypes, the case/control omnibus test is an H-1 degree of freedom test.

## Results

### Associations of *SLC6A20* SNPs and haplotypes with HSCR

**Figure 1:**
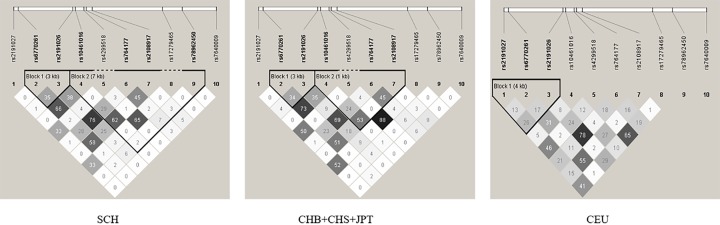
The LD patterns of the susceptibility SNPs in different populations

Ten SNPs in *SLC6A20* were selected for replication in Southern Chinese using 1470 cases and 1473 controls as shown in Table 1. SNP rs7640009 showed significant association with HSCR (*P*=3.56E-07). Inconsistent with the report by Lee et al. [17], we failed to replicate the association of the other nine SNPs (rs2191027, rs6770261, rs2191026, rs10461016, rs4299518, rs764177, rs2108917, rs17279465 and rs78962450) with HSCR. In order to identify the independence of variants of *SLC6A20* associated with HSCR, we examined the LD (*r^2^*) patterns of the selected ten SNPs among Southern Han Chinese (SCH) from the present study, East Asian population including CHB, Han Chinese in Singapore (CHS) and Japanese in Tokyo (JPT), and Utah residents with ancestry from northern and western Europe (CEU) from 1000 Genome project (Figure 1). Similar LD patterns were observed between SCH population and East Asian population from public data. We also found more complex LD structures in Asians compared with CEU population, two similar LD blocks were found in SCH population and East Asian population but only one in those in CEU. SNP rs6770261 and rs2191026 belonged to the same LD block in SCH population and East Asian population with low LD (*r^2^* > 0.33), but their LD (*r^2^* < 0.20) was weaker in CEU population, reflecting different genetic backgrounds of disease.

**Table 1:** Replication results of SNPs on SLC6A20 selected through 1470 cases and 1473 controls in South Chinese population

CHR	SNP	Gene	Function	POS	A1/A2	F_A	F_U	*P*	OR
3	rs2191027	*SLC6A20*	Intronic	45762899	T/C	0.015	0.012	0.312	1.26 (0.80–1.99)
3	rs6770261	*SLC6A20*	Intronic	45763242	T/C	0.217	0.204	0.206	1.09 (0.96–1.23)
3	rs2191026	*SLC6A20*	Intronic	45767003	T/C	0.431	0.410	0.098	1.09 (0.98–1.21)
3	rs10461016	*SLC6A20*	Intronic	45767209	C/T	0.232	0.218	0.207	1.08 (0.96–1.23)
3	rs4299518	*SLC6A20*	Intronic	45767781	C/T	0.036	0.038	0.748	0.96 (0.73–1.26)
3	rs764177	*SLC6A20*	Intronic	45767957	A/C	0.489	0.468	0.107	1.09 (0.98–1.21)
3	rs2108917	*SLC6A20*	Intronic	45768351	A/G	0.311	0.298	0.272	1.07 (0.95–1.19)
3	rs17279465	*SLC6A20*	Intronic	45773256	G/A	0.036	0.035	0.899	1.02 (0.77–1.35)
3	rs78962450	*SLC6A20*	Intronic	45774615	C/T	0.076	0.080	0.559	0.94 (0.78–1.14)
3	rs7640009	*SLC6A20*	Intronic	45776980	G/A	0.118	0.165	**3.56E-07**	0.68 (0.59–0.79)

Values in bold represent *P*<0.05 statistical significance.

Abbreviations: CHR, chromosome; POS, nucleotide position. A1/A2 indicates the risk allele and protective allele to disease; F_A/F_U indicates risk allele frequency of the SNP in cases or controls. The calculation of odds ratio (OR) is based on the risk allele of each SNP.

Haplotype analysis was also performed using logistic regression on the ten SNPs. As shown in Table 2, five haplotypes (ht2, ht4, ht9, ht10 and ht12) were identified as associated with HSCR. Intriguingly, except for ht4 (C-T-T-C-T-A-A-A-T-A) considered as a risk haplotype (*F_A* = 0.184, *F_U* = 0.161, *P*=0.021), the rest were confirmed as protective haplotypes. Ht9 (C-C-C-T-T-C-G-A-T-G) presented the strongest effect size in reducing the risk of HSCR than each of the others (*F_A* = 0.021, *F_U* = 0.047, *P*=8.90E-08).

**Table 2:** Haplotypic analysis of the ten SNPs on SLC6A20 showing evidence of associations with HSCR

	SNP1	SNP2	SNP3	SNP4	SNP5	SNP6	SNP7	SNP8	SNP9	SNP10	F_A	F_U	*P*
ht1	C	C	C	T	T	C	G	A	T	A	0.429	0.424	0.718
ht2	C	C	C	T	T	A	A	A	T	A	0.009	0.017	**6.41E-03**
ht3	C	C	T	C	T	A	A	A	T	A	0.034	0.028	0.246
ht4	C	T	T	C	T	A	A	A	T	A	0.184	0.161	**0.021**
ht5	T	C	T	T	C	A	G	G	T	A	0.014	0.011	0.293
ht6	C	C	C	T	T	A	A	A	T	G	0.047	0.041	0.332
ht7	C	T	T	T	C	A	A	G	T	A	0.014	0.017	0.414
ht8	C	C	T	T	T	A	G	A	T	A	0.156	0.142	0.132
ht9	C	C	C	T	T	C	G	A	T	G	**0.021**	**0.047**	**8.90E-08**
ht10	C	C	T	T	T	A	G	A	T	G	0.012	0.023	**1.40E-03**
ht11	C	C	C	T	T	C	G	A	C	A	0.066	0.065	0.906
ht12	C	T	T	C	T	A	A	A	T	G	0.015	0.024	**0.015**

Values in bold represent *P*<0.05 statistical significance.

Abbreviation: ht, haplotype. F_A/F_U indicates risk allele frequency of the SNP in cases or controls. The calculation of odds ratio (OR) is based on the risk allele of each SNP.

### Stratification analysis of *SLC6A20* gene polymorphisms with HSCR susceptibility

Regarding the length of the affected segment, HSCR patients were classified into diverse clinical subgroups. As shown in Table 3, association analysis of the selected ten SNPs on *SLC6A20* with the risk of different subclinical types of HSCR patients including SHSCR, LHSCR, TCA and TIA was performed. Slightly inconsistent with the previous study [17], we found SNP rs7640009 was more likely to affect SHSCR patients with the greatest effect size (*P*=8.88E-07) comparing with the LHSCR patients (*P*=0.005). Six SNPs (rs2191027, rs6770261, rs2191026, rs10461016, rs4299518 and rs764177) were observed to be related to the risk of severe subgroup of HSCR including LHSCR, TCA and TIA, respectively, while none of them seemed to be associated with HSCR as an entirety (Table 1). Especially for SNP rs2191027, the risk of TCA elevated two magnitudes in terms of *P*-value (*P*=1.09E-04) comparing with others, even at disease status quantitative association analysis (*P*=3.19E-04). SNP rs4299518 also principally affected TCA patients (*P*=0.33 and *P*=0.017 at disease status quantitative association analysis). As for SNPs rs6770261 (*P*=0.008), rs2191026 (*P*=0.046) and rs10461016 (*P*=0.012), they significantly increased the risk of TIA. SNP rs764177 was found to mainly affect LHSCR (*P*=0.025). We failed to replicate any association of SNPs rs2108917, rs17279465 or rs78962450 with stratified subclinical types.

**Table 3:** Association of SLC6A20 SNPs with HSCR patients analyzed by subphenotypes stratification

SNP	S-HSCR			L-HSCR			TCA			TIA			Disease status Quantitative Association *P* value
	F_A	F_U	*P*	F_A	F_U	*P*	F_A	F_U	*P*	F_A	F_U	*P*	
rs2191027	0.011	0.012	0.873	0.020	0.012	0.090	0.047	0.012	1.09E-04	NA	0.015	0.762	3.19E-04
rs6770261	0.218	0.204	0.240	0.224	0.204	0.267	0.201	0.204	0.939	0.667	0.217	0.008	0.997
rs2191026	0.425	0.410	0.275	0.448	0.410	0.085	0.421	0.410	0.778	0.833	0.429	0.046	0.599
rs10461016	0.231	0.218	0.300	0.241	0.218	0.236	0.228	0.218	0.773	0.667	0.232	0.012	0.708
rs4299518	0.032	0.038	0.266	0.041	0.038	0.698	0.071	0.038	0.033	NA	0.036	0.634	0.017
rs764177	0.480	0.468	0.418	0.519	0.468	0.025	0.494	0.468	0.530	0.833	0.488	0.091	0.208
rs2108917	0.306	0.298	0.565	0.332	0.298	0.113	0.298	0.298	0.992	0.667	0.311	0.060	0.522
rs17279465	0.033	0.035	0.649	0.043	0.035	0.367	0.047	0.035	0.411	NA	0.036	0.636	0.171
rs78962450	0.079	0.080	0.959	0.079	0.080	0.913	0.047	0.080	0.122	NA	0.077	0.480	0.263
rs7640009	0.115	0.165	8.88E-07	0.118	0.165	0.005	0.135	0.165	0.310	NA	0.118	0.370	0.505

F_A/F_U indicates risk allele frequency of the SNP in cases or controls. The *P*-value indicates the significance in different subclinical manifestation. The calculation of odds ratio (OR) is also based on the risk allele of each SNP.

Since HAEC was the most common serious complication of HSCR, the relationship of the ten selected SNPs and HAEC pre-/post-operation was investigated by patient-only analysis as presented in Table 4. SNP rs2191026 was found to be associated with the susceptibility of HAEC after surgery (odds ratio (*OR*) = 0.79, *P*=0.04).

**Table 4:** Patient-only associations of SLC6A20 SNPs with HSCR Enteritis stratification

CHR	SNP	A1/A2	Enteritis before operation		Enteritis after operation	
			*P*	OR	*P*	OR
3	rs2191027	T/C	0.340	1.55 (0.63–3.84)	0.763	0.85 (0.29–2.46)
3	rs6770261	T/C	0.753	0.96 (0.74–1.24)	0.620	0.94 (0.72–1.22)
3	rs2191026	T/C	0.169	0.86 (0.69–1.07)	0.040	0.79 (0.63–0.99)
3	rs10461016	C/T	0.748	0.96 (0.74–1.24)	0.639	0.94 (0.72–1.22)
3	rs4299518	C/T	0.367	1.29 (0.74–2.23)	0.167	0.63 (0.32–1.22)
3	rs764177	A/C	0.076	0.82 (0.66–1.02)	0.232	0.87 (0.70–1.09)
3	rs2108917	A/G	0.230	0.86 (0.68–1.10)	0.723	0.96 (0.76–1.22)
3	rs17279465	G/A	0.719	1.11 (0.63–1.97)	0.271	0.70 (0.37–1.33)
3	rs78962450	C/T	0.067	1.44 (0.97–2.14)	0.223	1.28 (0.86–1.91)
3	rs7640009	G/A	0.805	0.96 (0.68–1.35)	0.279	1.21 (0.86–1.69)

Abbreviation: CHR, chromosome. A1/A2 indicates the risk allele and protective allele to disease. The calculation of OR is based on the risk allele of each SNP.

### Expression quantitative trait loci analysis of *SLC6A20* SNPs

**Figure 2:**
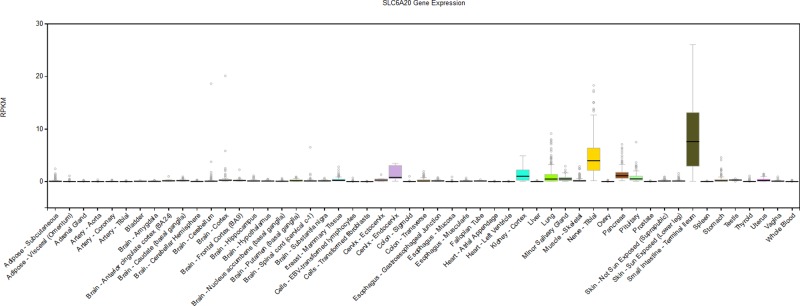
SLC6A20 expression patterns in different tissues extracted from GTEx portal The GTEx Portal (GTEx Consortium, 2015) (GTEx Analysis Release v.6, http://www.gtexportal.org/) was used to analyze the association of SNPs with genes.

In order to examine the expression quantitative trait loci (eQTL) association between the ten selected SNPs and *SLC6A20* and other genes, two datasets, from Westra et al. [18] and Raj et al. [19], were investigated. In the first study, a meta-analysis was performed to assess the relationship between SNPs and gene expression, using peripheral blood samples of 5311 individuals with replication in 2775 individuals. In the second study, the researchers examined the correlation of SNPs to gene expression using purified CD4^+^ T cells and monocytes in a multi-ethnic cohort of 461 healthy individuals. As exhibited in Table 5, we failed to find out the potential regulatory role of the replicated SNP rs7640009 in the current dataset. Considering the haplotypic associations with disease, we also examined the eQTL with other SNPs. Interestingly, seven of the ten SNPs recruited in the present study were identified as eQTL on *SLC6A20*, among which six SNPs (rs2191027, rs2191026, rs10461016, rs4299518, rs764177 and rs17279465) were found to be also correlated with the expression of *SACM1L*, SNP rs2191026 was connected with the expression of *LARS2* as well. SNP rs2108917 was detected to be related to the expression of *LIMD1* (Table 5). These results might suggest functional connections among these genes with HSCR, although further validation is required. *SLC6A20* were found to be expressed at the high level both in the terminal ileum and tibial nerve in its expression patterns in different tissues extracted from the Genotype-Tissue Expression (GTEx) portal (Figure 2), indicating that *SLC6A20* might play an analogic role in intestine tissue and nerve tissue.

**Table 5:** The eQTL evidence of the SNPs on SLC6A20

SNP	Cell_type	*Cis/Trans*	Study	*P*-value	Gene 1	*P*-value	Gene 2	PMID
rs2191027	PBMC	*Cis*	Meta	8.61E-24	*SACM1L*	-	-	24013639
rs2191026	PBMC	*Cis*	Meta	1.33E-12	*SACM1L*	1.13E-04	*LARS2*	24013639
rs10461016	CD4T	*Cis*	Meta	7.76E-07	*SACM1L*	-	-	24786080
rs4299518	PBMC	*Cis*	Meta	5.08E-09	*SACM1L*	-	-	24013639
rs764177	PBMC	*Cis*	Meta	1.03E-07	*SACM1L*	-	-	24013639
rs2108917	CD4T	*Cis*	Meta	8.05E-08	*LIMD1*	-	-	24786080
rs17279465	PBMC	*Cis*	Meta	4.02E-08	*SACM1L*	-	-	24013639

Abbreviations: *Cis*, cis-acting element; *Trans*, trans-acting element. Expression level of *SLC6A20* gene was obtained from public RNA-seq data resource and genotypes of SNPs around SLC6A20 were obtained from the 1000 Genome project website (http://browser.1000genomes.org/).

## Discussion

HSCR is well-known as a complex polygenic disease, whose etiology and pathogenesis are still unclear at present. More than 15 genes such as *RET* [12], *EDNRB* [13], *GDNF* [14] and *SOX10* [15] are related to the disease in previous studies, most of which however are of limited quantities or pedigree studies, and few of which have been conducted on a large number of sporadic cases. Owing to the application of GWAS, an increasing number of studies have detected more new disease-causing genes. For example, Kim’s study [16] has identified the new susceptibility locus of HSCR. Then, replicated study [17] with only 187 cases and 283 controls have been carried out to confirm the associations between 13 SNPs on in *SLC6A20* and HSCR. Fortunately, our present study has replicated Lee et al.’s [17] work as well using the largest amount of sample size with 1470 cases and 1473 controls. The association between ten selected SNPs and HSCR were investigated. Our results showed the significant association between only SNP rs7640009 and HSCR and failed to reveal the associations between the other nine SNPs (rs2191027, rs6770261, rs2191026, rs10461016, rs764177, rs2108917, rs17279465, rs78962450 and rs4299518) and HSCR, which was inconsistent with Lee et al.’s [17] study. (Table 1). Through the further clinical stratification analysis, we found that SNP rs7640009 was more likely to affect SHSCR patients comparing with the LHSCR patients, and six SNPs (rs2191027, rs6770261, rs2191026, rs10461016, rs4299518 and rs764177) were observed to be associated with the risk of severe subgroups of HSCR, only four (SNPs rs6770261, rs2191026, rs10461016 and rs764177) of which showed correlations with LHSCR in Lee et al.’s study [17]. Three SNPs (rs6770261, rs2191026 and rs10461016) were found to increase the risk of TIA, but TIA patients were even not mentioned in the previous study (Table 3). This inconformity in our study and Lee et al.’s [17] study was probably because of ethnic diversity or sample size, which might be reflected by different genetic backgrounds.

Nevertheless, our results may have implications for the genetic counseling of HSCR in southern China. Focusing on the examined SNPs of *SLC6A20*, some risk alleles of SNPs may raise the risk of HSCR or some clinical subtypes of HSCR. For instance, the risk allele of SNP rs7640009 can increase the risk of HSCR, SHSCR and LHSCR. Patients with risk alleles of SNPs rs6770261, rs2191026 or rs10461016 are likely to be affected by TIA, the rarest and severest type of HSCR. Besides, risk alleles of SNPs including rs764177, rs2191027 and rs4299518 may raise the incidence rates of LHSCR and TCA, respectively. Meanwhile, the other three SNPs rs2108917, rs17279465 and rs78962450 will be of no significance to the diagnosis of HSCR. The present study can still provide some help for people in need although only part of *SLC6A20* is involved.

*SLC6A20* gene encodes SIT1, which is a member of the SLC6 Na^+^- and Cl^−^-dependent neurotransmitter transporter family [20]. Rat SIT1 mRNA is expressed in epithelial cells of colon, duodenum, ileum, jejunum, stomach and cecum [21]. Alterations occurred in the balance of expression of neurotransmitters in the colon proximal to the aganglionic region of animal model [22]. Our study discovered that TIA was more associated with *SLC6A20* SNPs than SHSCR. These results suggest that the dysfunction of *SLC6A20* variations was likely to affect its interactions with other risk factors of HSCR in the extent of the aganglionosis during the development of enteric nervous system (ENS), Moreover, public RNA-seq data revealed the evidence of the eQTL of seven selected SNPs on *SLC6A20*, and the impact of SNPs on the expressions of genes around *SLC6A20* (Table 5). *SACM1L* is one of the stable and coordinated gene transcriptional networks regulating brain phosphoinositide metabolic pathways during the development and aging of human beings [23]. Bipolar disorder and schizophrenia may be pathophysiologically attributed to the accumulated 3243A>G mutation of *LARS2* in the brain [24]. *SACM1L* and *LARS2* may have some similar effect during the development of ENS and brain since ENS is composed of a complex neurological structure resembling central nervous system (CNS). Involved in the Hippo signaling pathway, whose network integrated a wide range of biochemical and biomechanical cues to modulate outraged growth and cell fate [25], *LIMD1* was also proven to exert an influence on cell migration [26] that was essential during the development of ENS. Since the four mentioned were in the same region of Chromosome 3, the interactions between among them could cast light on interpreting how *SLC6A20* boosted the predisposition of HSCR, a disorder caused by the development of ENS.

HAEC is the most invalid and life-threatening complication in HSCR patients [27]. It is still unclear about the potential mechanisms of enterocolitis. Reportedly, the reduced expression of *CDX2* gene in mucosa may be associated with the development of HAEC [28]. As a member of the solute carrier family and a target gene of *CDX2, SLC7A7* can be activated during the proliferation of normal intestinal epithelial cells induced by *CDX2* [29]. Considering the interaction of the solute carrier family, *CDX2* and HAEC and *SLC6A20* polymorphisms may be likely to boost the have the development of HAEC.

However, several limitations should be noted in our study which is based on a tertiary medical center in spite of its large sample size. TIA cases are insufficient due to the scarcity of conditions, thereby requiring other cohorts in China to confirm the associations in the present study. Moreover, the present study made a bioinformatics analysis to only estimate the potential functions of *SLC6A20* SNPs, calling for further functional evaluations.

## Conclusion

Our study verified the important role of SNP rs7640009 in increasing the risk of HSCR. The haplotype analysis revealed that the C-T-T-C-T-A-A-A-T-A haplotype in *SLC6A20* might be a genetic susceptibility factor for HSCR. The expression eQTL of the SNPs on *SLC6A20* and their different association patterns in different subclinical groups suggested a gene–dose effect in the extent of the aganglionic segment during the development of ENS. We found that the regulation of tissue-specific genes should be taken into consideration to study the effects of disease-related SNPs. As far as we know, our study is the first one to report the association between *SLC6A20* polymorphism and HAEC susceptibility and plays a functional role for in small intestine-related SNPs as gene regulators, although further studies remain to be conducted.

## Abbreviations

CEU: Utah residents with ancestry from northern and western Europe
CHB: Han Chinese in Beijing
ENS: enteric nervous system
eQTL: expression quantitative trait loci
HAEC: Hirschsprung’s disease-associated enterocolitis
HSCR: Hirschsprung’s disease
HWE: Hardy–Weinberg equilibrium
LD: linkage disequilibrium
LHSCR: long-segment HSCR
SCH: Southern Han Chinese
SHSCR: short-segment HSCR
SNP: single nucleotide polymorphism
TCA: total colon aganglionosis
TIA: total intestine aganglionosis
